# Heterotopic Pregnancy After Ovulation Induction by Clomiphene Citrate: A Case Report

**DOI:** 10.7759/cureus.101281

**Published:** 2026-01-11

**Authors:** Nada Douraidi, Hassnaa Sarhane, Fatima Zahra Belouaza, Soukaina Mouiman, Aziz Baidada

**Affiliations:** 1 Gynecology-Obstetrics and Endoscopy Department, Maternity Souissi, University Hospital Center IBN SINA, Rabat, MAR

**Keywords:** clomiphene citrate, heterotopic pregnancy (hp), ovulation induction, salpingotomy, ultrasonographic diagnosis

## Abstract

Heterotopic pregnancy (HP), when intrauterine and ectopic pregnancies occur together, is uncommon but dangerous. While rare in spontaneous conception, the risk increases with fertility treatments like clomiphene citrate (CC). These cases are easily missed because doctors may be reassured by the intrauterine pregnancy and overlook the ectopic one. We describe a 31-year-old woman with primary infertility who conceived following clomiphene-induced ovulation. At seven weeks, she developed pelvic pain and vaginal bleeding. She was hemodynamically unstable with tachycardia and low blood pressure. Ultrasound showed an intrauterine sac alongside a left adnexal sac without fetal heartbeat, plus fluid in the pelvis indicating bleeding. We diagnosed HP and performed emergency laparotomy with left salpingectomy due to her unstable condition. She recovered well with first-trimester progesterone support and delivered vaginally at 40 weeks without complications. This case highlights how challenging HP can be to diagnose. Even when an intrauterine pregnancy is visible, clinicians should consider HP in patients with pelvic pain and fertility treatment history. Clomiphene increases this risk even without other predisposing factors. Ultrasound must include careful adnexal examination to avoid missing the diagnosis. Hemodynamically unstable patients need immediate surgery to control bleeding and protect the intrauterine pregnancy. With timely recognition and proper management, including progesterone support, good outcomes are possible. Vigilance is essential for all patients undergoing ovulation induction.

## Introduction

Heterotopic pregnancy (HP) occurs when a woman carries both an intrauterine and an ectopic pregnancy at the same time [1-2]. This is quite rare in natural pregnancies, happening in roughly one out of every 30,000 cases [3-4]. However, as fertility treatments have become more common, we are seeing HP more frequently - now affecting about one in 3,900 pregnancies among women using assisted reproductive technologies (ARTs) or ovulation-inducing medications [4-5].

Women who develop HP typically share the same risk factors as those prone to ectopic pregnancies. A history of pelvic inflammatory disease, prior ectopic pregnancies, previous tubal surgeries, or pelvic adhesions all increase vulnerability. Fertility medications like clomiphene citrate (CC) and gonadotropins can further elevate this risk, particularly when there's pre-existing damage to the fallopian tubes [6].

The challenge with HP lies in catching it early. Once physicians identify an intrauterine pregnancy on ultrasound, they often feel reassured and may not think to look for an additional ectopic pregnancy happening simultaneously. While transvaginal ultrasound remains our primary diagnostic tool, it doesn't always pick up HP in the early weeks, leading to missed diagnoses [7]. Unfortunately, this diagnostic delay can result in dangerous complications, including ruptured tubes and significant internal hemorrhage.

Treatment focuses on eliminating the ectopic pregnancy while safeguarding the healthy intrauterine one. Surgeons most commonly perform laparoscopic salpingectomy to achieve this [8].

We report here a case of HP that developed after CC was used for ovulation induction. Our case underscores why clinicians must remain alert to the possibility of HP, especially when patients have undergone fertility treatments.

## Case presentation

We report the case of a 31-year-old woman, with no significant past medical history, married for five years with no known consanguinity, and no history of contraception use. She presented with primary infertility of five years’ duration.

This was her first pregnancy (G1P0), estimated at seven weeks of gestation, achieved following ovulation induction with CC.

She presented to the emergency department with a three-day history of left-sided pelvic pain, associated with minimal dark vaginal bleeding.

On clinical examination, the patient showed signs of hemodynamic instability with tachycardia at 105 beats per minute and hypotension (blood pressure 90/60 mmHg). Abdominal examination revealed localized tenderness in the left iliac fossa.

Gynecological examination showed minimal dark bleeding originating from the endocervix.

**Figure 1:**
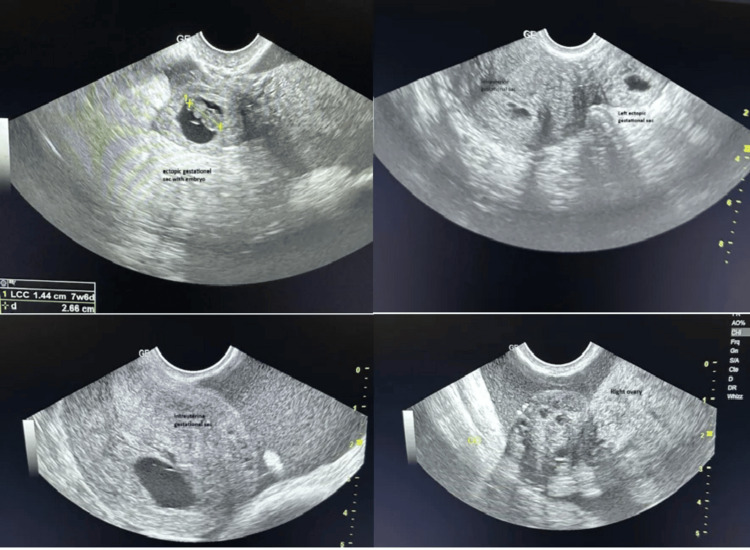
Transvaginal ultrasonography showing heterotopic pregnancy at seven weeks + six days

Transvaginal pelvic ultrasound revealed an anteverted, anteflexed uterus of increased size, with a thickened endometrium and the presence of an intrauterine gestational sac measuring 6 × 5 cm containing a yolk sac but no visible embryo. In addition, a second gestational sac was identified in the left adnexal region, containing an embryo with a crown-rump length (CRL) corresponding to seven weeks and six days of gestation, but with no detectable cardiac activity. Signs of rupture were present, including a small amount of free fluid in the pouch of Douglas (Figure 1).

**Figure 2:**
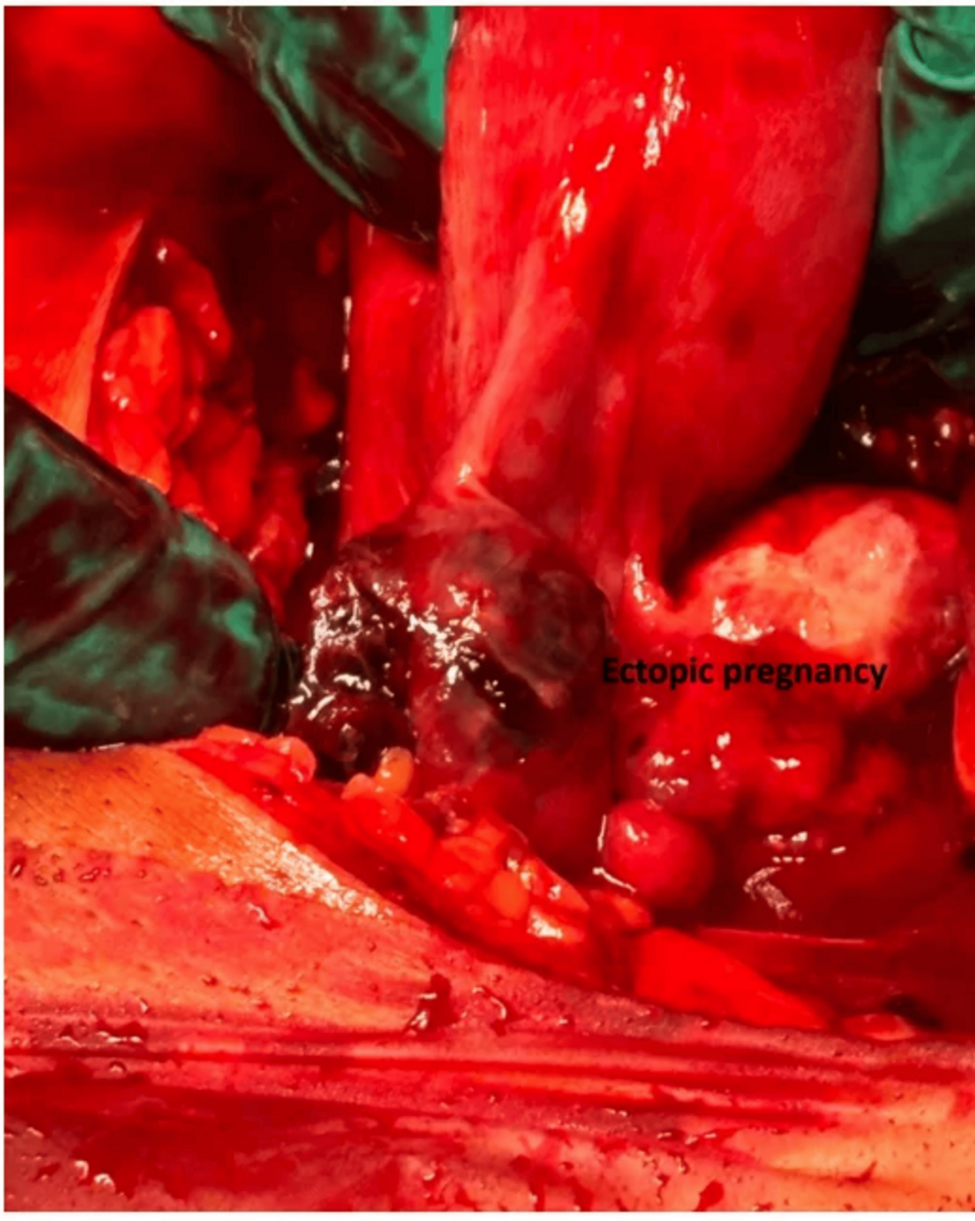
Intraoperative view of a left-sided tubal ectopic pregnancy coexisting with an intrauterine gestation.

A diagnosis of HP was made. Given the patient’s hemodynamic instability with tachycardia and hypotension, immediate surgical intervention was required. After careful counseling and obtaining written informed consent, an emergency laparotomy with left salpingectomy was performed rather than laparoscopy due to the hemodynamic compromise (Figure 2). During surgery, a hemoperitoneum of 50 mL was found and evacuated. Both ovaries were found to be normal.

Postoperative recovery was uneventful. The patient was discharged on postoperative day 3 in stable condition. Throughout the first trimester, she received progesterone supplementation to support the intrauterine pregnancy. Regular antenatal follow-up was maintained, and the pregnancy progressed normally without complications. At 40 weeks and three days of gestation, she delivered vaginally a healthy newborn weighing 3,200 grams with Apgar scores of 9 and 10 at one and five minutes, respectively. Both mother and baby had an uncomplicated postpartum course.

## Discussion

When both an intrauterine and ectopic pregnancy develop at the same time, we call this HP, a relatively uncommon occurrence. Natural conception sees this combination happen once in approximately 30,000 pregnancies. However, fertility treatments change these odds considerably: in vitro fertilization increases the rate to about one in 100, while CC brings it to roughly one in 900 [9].

The connection between CC and multiple pregnancies has been recognized for decades. In fact, Payne's team first reported a heterotopic case following clomiphene treatment in 1971. Since clomiphene became a frontline option for helping women ovulate, physicians have documented numerous similar occurrences [10-13].

What makes ovulation induction particularly relevant here? The process itself may contribute to the problem beyond typical ectopic risk factors. Clomiphene triggers elevated estrogen production, which can interfere with normal egg transport timing through the fallopian tubes. When an egg moves more slowly than usual, fertilization might occur before it reaches the uterus, setting the stage for tubal implantation.

Identifying HP presents real clinical challenges. The symptoms do not necessarily stand out; they can mirror either uncomplicated pregnancy or isolated ectopic pregnancy. Here is the problem: seeing a gestational sac in the uterus tends to put clinicians at ease, sometimes causing diagnostic delays. Consider this data point among 139 documented HP cases, ultrasound identified only 80 before surgery; the other 59 went unrecognized until doctors were already operating [14]. Complicating matters further, HP can easily be mistaken for conditions like a bleeding corpus luteum alongside normal pregnancy, or ovarian hyperstimulation syndrome.

While transvaginal ultrasound serves as our primary diagnostic method, its detection rate for HP sits at just 33% [15]. Given this limitation, the National Guideline Alliance advises that examining the adnexal regions should be standard practice during all first-trimester scans [16]. Skipping this step can mean missing HP until serious complications develop, such as internal hemorrhage from a ruptured tube.

Treatment strategies vary based on the patient's condition [17]. For stable, symptom-free women, careful observation might be appropriate, though rupture remains a concern requiring vigilant ultrasound follow-up [18]. Surgical intervention-whether minimally invasive or open-becomes imperative when patients are unstable or rupture seems likely. Our patient presented with cardiovascular instability, including elevated heart rate and low blood pressure, which led us to choose open surgery over laparoscopy for faster hemorrhage control. Depending on the ectopic location, surgeons might remove the entire affected tube or perform a more conservative procedure to extract only the pregnancy tissue. First-trimester progesterone therapy may benefit the continuing intrauterine gestation after removing the ectopic component. As our experience illustrates, combining appropriate surgical technique with hormone supplementation can result in successful full-term delivery [19]. An alternative approach uses ultrasound-guided needle aspiration of the ectopic gestational sac, potentially combined with non-teratogenic agents (methotrexate is contraindicated because it causes birth defects). This less invasive method only applies when visualization is excellent and hemodynamic parameters remain normal [19]. The bottom line: treatment must be tailored to each patient, and collaborative care among specialists consistently produces superior outcomes.

## Conclusions

While HP does not happen often, it creates real difficulties for clinicians trying to diagnose and manage it especially when fertility treatments are involved. Our case highlights an important lesson: doctors cannot let their guard down just because they have spotted a pregnancy in the uterus. The presence of an intrauterine gestation should not provide false comfort.

Ultrasound through the vaginal approach continues to be our most valuable diagnostic tool, but here is the catch, it only works well when doctors carefully and methodically check the areas around the ovaries and tubes. What is particularly noteworthy is that HP can develop even in women who do not fit the typical risk profile, particularly after using medications like CC to stimulate ovulation.

The key takeaway? Catching this condition quickly and correctly can make all the difference. Missing the diagnosis can lead to dangerous complications, while identifying it promptly gives us the best shot at protecting the healthy pregnancy developing in the uterus
